# Doctors as research participants - what is in it for them? A qualitative study of international medical graduates

**DOI:** 10.1186/s12909-025-07468-1

**Published:** 2025-07-01

**Authors:** Sunita Joann Rebecca Healey, Kristy Fakes, Bunmi S Malau-Aduli, Balakrishnan R Nair

**Affiliations:** University of Newcastle- School of Medicine and Public Health, Callaghan, NSW 2308 Australia

**Keywords:** Health services research, Altruism, Confidentiality, Volunteer, Motivation, Disclosure, Participation

## Abstract

**Background:**

Doctors are key players in the healthcare system. Their first-hand experience in the workplace may provide researchers with special insight into health service gaps and solutions. However, doctors are typically time poor with high professional demands. Little is reported about how to best engage doctors in research as participants. International medical graduates (IMGs) are a special cohort of doctors with unique challenges, whose experiences and perceptions have been scarcely voiced within research to date.

**Methods:**

We conducted a qualitative study to explore IMG motives of participation in research. The study was conducted in February to April 2024 and comprised of semi-structured individual interviews with IMGs based in Australia. The interviews followed an online national survey which had been conducted the previous year. Recruited interview participants were primarily drawn from participants from the preceding survey who had indicated interest in participation in the follow-up qualitative study, in addition to any participants indicating interest via snowballing. Participants were eligible for inclusion if they were living or working in Australia, with a primary medical qualification from overseas, irrespective of current employment status. Data was audio-recorded and thematically analysed.

**Results:**

IMGs (36) were individually interviewed by telephone (21), teleconference (14) and face-to-face (1). We identified three major themes and four subthemes: (1) Research is considered a safe and trusted tool; 1a) Research provides an opportunity to voice, broadcast and enact change, 1b) Safe and anonymous sharing of personal stories and experiences is therapeutic; (2) Personal relevance and interest motivates participation; 2a) Passion for the research topic drives participation, 2b) Comradery and empathy for peers convicts participants to advocate for others through engagement (3) Respect for Research drives engagement.

**Conclusion:**

IMGs have a variety of personal motives for participating in research. Understanding these motives can be useful for researchers seeking to recruit such doctors for research in the future.

**Supplementary Information:**

The online version contains supplementary material available at 10.1186/s12909-025-07468-1.

## Background

It is imperative that health services are innovative and adaptive to the evolving needs of consumers and providers of healthcare [1]. Consumer engagement coupled with clinical leadership and evidence-based practice are features of innovative design and planning of healthcare services worldwide [2, 3]. As integral providers within health services, doctors may possess special insights into the inner mechanics of health system environments, which may provide opportunities for change in health policies and work conditions. Therefore, their participation is important to gain medical viewpoints into healthcare, and also to explore research gaps involving this workforce.

Several sporadic studies from the perspective of doctors as participants have been published, predominantly exploring the doctor-patient relationship [4]doctors as patients [5, 6] perceptions about doctors roles [7, 8] and workplace issues such as fatigue in junior doctors [9]training and careers [10, 11] and inequitable treatment [12, 13].

A special group of doctors whose experiences and perceptions are scarcely voiced in research, are international medical graduates (IMGs). IMGs have obtained their primary medical qualification (PMQ) outside the country they are currently based in [14]. In Australia, it is estimated that a third of working doctors are IMG [15]. This is similar to rates in other developed countries [16, 17]. With increasing globalisation of the medical workforce, there is growing interest pertaining to experiences, training and support of IMGs. In 2023–2024, as part of a PhD research project, we undertook an anonymous, national Australian study exploring IMG lived experiences, personal health outcomes and supports. To date, two papers arising from this work has been published, detailing work-related inequitable treatment reported by the study population [13, 18]. 

As researchers, we were overwhelmed by the uptake of our study by IMGs Australia-wide; a total of 286 IMG survey participants, from 46 different PMQ countries and a variety of clinical and non-clinical backgrounds participated. The embrace of IMGs in Australian research has also been observed 20 years ago, with production of the ‘Lost in the Labyrinth Report’ [19]. The Report was the culmination of recommendations resulting from an Australian national parliamentary inquiry into registration processes and support for overseas trained doctors. The Report canvassed 109 submissions provided by IMGs who shared details of their personal stories. Interestingly, a third requested anonymity for fear of repercussion. Given the time-poor nature of doctors, together with highly demanding work commitments of the medical profession, it follows that recruiting doctors as participants in studies may pose difficulty for prospective researchers. Although doctors have been studied as conductors of research [20]there is an absence of published literature describing the role of doctors as research participants themselves.

We were therefore interested to explore the generosity of our study participants, their willingness to freely volunteer their time for our study and their motives to share their personal experiences. The data in this study may assist prospective researchers interested in recruiting doctors as participants. This may in turn broaden capture of valuable data in the space of health services and medical education.

## Methods

### Study design and setting

By undertaking a qualitative study with semi-structured interviews, we used a phenomenological approach to explore underlying motives of IMGs participating in a larger overarching research study. The research study was granted full ethics approval, considered low risk research: Human Ethics Advisory Panel at the University of Newcastle H2022-0392 with Access Request approved through Hunter New England Health Human Research Ethics Committee (AR20230405_Nair) and Central Coast Health Human Research Ethics Committee (0323–024 C). Interviews were conducted between February to April 2024.

### Participants, recruitment and sample size

Participants were IMGs living in Australia, irrespective of employment status. IMGs were excluded if they were casually visiting Australia for non-work purposes (e.g., recreational holiday). Interview participants were primarily drawn from participants who had expressed interest in the preceding survey. No financial incentives were offered or provided for participation in the research. Interview sample size was determined by the number of written consent forms returned to the primary researcher within the three-month interview study phase period.

### Data tools, collection and handling

Participants were interviewed individually by SJRH by telephone, teleconference, or face-to-face. Time-zones were considered for participants who were located in different states or territories to the interviewer. The full qualitative study comprised of six semi-structured interview questions. Interview time was forewarned and estimated at 30–60 min. The final interview question, pertinent to this paper, explored participant motivations for participating in the study interview [see supplemental material 1]. Two data recorders were used to capture audio data, which were transcribed within 48 h by the primary researcher (SJRH).

#### Analysis

Thematic analysis was undertaken as per Clarke and Braun’s guidelines [21]. SJRH created preliminary mind-maps of common ideas surfacing from the interviews. These ideas were explored and discussed with senior researchers, KF and BSMA.

Facilitated by NVivo [22]the coding tree was co-created by SJRH and KF. The researchers met weekly over several weeks to discuss and refine codes, categories and themes using an iterative process.

Word-versions of the transcripts were imported into NVivo and read several times by SJRH ± KF independently, to familiarise themselves with the data. Overall, SJRH and KF dual coded four (i.e., 10% of all) transcripts, to check accuracy of matching. Any disagreements were discussed and reviewed against the data and study aims. BSMA was assigned to review any unsolvable coding disagreements between SJRH and KF, but input was not required. SJRH analysed the remaining 90% of transcripts independently but regularly checked concerns and reviewed the coding with KF. Initial common codes were generated, and relevant sections of text from each manuscript were sorted accordingly. Codes were reviewed, looking for differences and similarities and were combined into categories encapsulating similar groupings. Overarching themes were first identified by SJRH and then discussed, reviewed and refined by KF and BSMA (a third researcher with expertise in methodology) for final decision making. Themes were further refined at meetings between SJRH, KF and BSMA following subsequent manuscript revisions.

#### Stance and reflexivity

We used a stance of constructivism which claims that knowledge is subjective and is constructed from individual, social perspectives [23]. It is not controllable and has to be explored [23]. The primary researcher (SJRH) is an IMG, person of colour, and a clinical doctor of twenty years. Several procedures were undertaken to reduce the risk of implicit bias and assert research integrity, such as log booking, regular debriefing and data review with non-medical research team members (KF and BSMA) and exclusion of IMGs well-known to the primary researcher (SJRH).

## Results

### Participant details

The 36 interviewees came from a total of 16 different PMQ countries; ranged from 27 to 60 years old (mean 40.7 years); 27 identified as female and nine identified as male. Interviewees were drawn from across Australia; lived experiences were reported from each state or territory. A wide range of clinical, training and demographic backgrounds were represented by the interview participants. Interviews were conducted individually by telephone (21/36); teleconference (14/36) and face-to-face (1/36). Over 28.7 h of recorded transcription time was gathered for analysis; averaging 47.8 min per participant (range: 15.33–88 min).

#### Themes

We identified three major themes and four subthemes: (1) Research is considered a safe and trusted tool.; 1a) Research provides an opportunity to voice, broadcast and enact change, 1b) Safe and anonymous sharing of personal stories and experiences is therapeutic (2) Personal relevance and interest motivates participation; 2a) Passion for the research topic drives participation, 2b) Comradery and empathy for peers convicts participants to advocate for others through engagement; (3) Respect for Research drives engagement. See Table 1 for full description of illustrative quotes.

**Table 1:** Illustrative quotes of identified themes

ILLUSTRATIVE QUOTES OF IDENTIFIED THEMES
**1. THEME 1: Research is considered a safe and trusted tool**
**1a) Research provides an opportunity to voice, broadcast and enact change**
***Opportunity to express voice and raise awareness***
“Noone addressing our issues, and no one even, and I say, no one care, despite they [are] saying that they do care…but they don’t…and just literally, we can’t speak up.” [Interviewee #20; PMQ: Egypt]
“There’s this amazing number of IMGs here. And maybe they feel their voice isn’t always heard. And this is a way of making your voice heard and hopefully helping the next person.” [Interviewee #16; PMQ: South Africa]
“As if you were coming to a disadvantaged community, and no one knows about them….and then you ask them, *well guys*,* how do you feel*,* do you have any problems? do you feel you are treated differently?* Definitely everyone will like to speak. So, this is what you did actually.” [Interviewee #03; PMQ: Sudan]
“But I couldn’t tell them all this bulls*t that I experienced. I never told anyone. I never told my parents. I never told [my ex-partner]. I couldn’t tell anyone. There was no one to tell. And…at the time, I didn’t even think I needed to complain about these things. I just thought this is what it’s supposed to be…” [Interviewee #32; Trinidad and Tobago]
“So, I hope that you… try to reach [out] about this to make a good paper…I think your research will help others even if they read your PhD thesis.” [Interviewee #17; PMQ: Egypt]
“I am interested in letting people know how I felt and how I would have done things differently…. like there will be more awareness about this” [Interviewee #10; PMQ: India]
***Hope for change***
” and if this [research] gets put out there in the public, or locally, maybe people will change.” [Interviewee #10; PMQ: India]
“Because I think that my experience should inform change, yeah? So, I think your way of writing over it, and doing an assessment of the experience… is one way of inducing change, so it’s not going to be so difficult for the next person who comes.” [Interviewee #31; PMQ Germany]
“Because if the outcome [research results] is [are] delivered to the system, and then they [institutions] know the outcome, and they [institutions] implement [change] into the system…and I’m hoping that helps to the other IMGs… yeah, that’s my hope, that’s why I’m participating.” [Interviewee #25; PMQ: China]
**1b) Safe and anonymous sharing of personal stories and experiences is therapeutic**
“…this is like talking to a psychiatrist to vent out your heart, without being judged, without being taken, without something being taken against you…[to] talk to somebody who could listen and wouldn’t hold anything against me.” [Interviewee #33; PMQ: India]
“I feel like [the interview] it’s like some sort of therapy at the same time- about the struggles I face… So that is probably why most of us would like to participate… to talk… about what we feel and what have we suffered.” [Interviewee #03; PMQ: Sudan]
“When I had down times… Where will I go to rant about all of this? As a normal human being, it’s very hard to bottle up that emotion.” [Interviewee #35; PMQ: Ireland]
“I think [IMGs participate] to vent. Because no one cares. No one cares about what you trying to achieve here, which is identify problems faced by IMGs in this country, and implement some sort of solution… I realised that a long time ago, that’s why I was never able to vent about it.” [Interviewee #32; PMQ: Trinidad and Tobago]
**THEME 2: Personal relevance and interest motivates participation**
**2a) Passion for the research topic drives participation**
“I feel quite strongly that we need to nurture and support IMGs. I just think- *wow*,* great! someone’s doing research into this*.” [Interviewee #21; PMQ: UK]
“I wouldn’t miss that opportunity [of being interviewed]. Because someone else is looking at our experience as a part of IMG. We are a specific mob. We…our experience is unique. So, someone looking into it as an interest, and then coming up with a strategy or a structure…framework to help IMGs to feel better, I think I’m in it” [Interviewee #34; PMQ: India].
**2b) Comradery and empathy for peers convicts participants to advocate for others through engagement**
“And I think there’s a kind of understanding, the hardship they have to go through.…I think there’s this sense of ‘we know what you’ve been through’….” [Interviewee #27; PMQ: South Africa]
“You are free to ask as many question as you want, and to take as much as you want from my time, because this service could be more helpful for people like myself…So I’m happy to help, anyway you like.” [Interviewee #20; PMQ: Egypt]
“So, I think that was the reason why I wanted to do it. To see if anything I said might help the next person have a slightly easier run.” [Interviewee #16; PMQ: South Africa]
“…I’d like to give back to someone one day. I’d like someone to say, *oh yeah I’m so glad I talked to [X] about it*,* because she showed me the right direction*. If I can make it a bit easier for somebody else, that would be great.” [Interviewee #27; PMQ: South Africa]
“I just feel like I should be sharing my experiences with other people, so it can prevent someone having the same pain as I did. Yeah, if I can save one IMG, or make them happy, or share my experience…then it’s worth it.” [Interviewee #28; India]
“I think the main reason why I wanted to participate is, I guess, because I’ve seen how hard it is for a lot of IMGs and how badly a lot of IMGs are treated.” [Interviewee #05; PMQ: Sweden]
**THEME 3: Respect for Research drives engagement**
“I would like to do a PhD, starting next year, and I think that I will have difficulty if I am trying to do a survey in recruiting people. So, I think it’s always good karma to participate in other people’s research.” [Interviewee #04; PMQ: UK]
“I acknowledge that my experience is not the standard experience. So, it was just to put that perspective.” [Interviewee #22; PMQ: Ireland]
“I have a thing that if there’s research, I will say yes…it’s just a personal thing. So, if I’m asked, I’ll participate.” [Interviewee #22; PMQ: Ireland]

## Theme 1: research is considered a safe and trusted tool

Participants described confidence in the ability of Research to disseminate rigorous findings in a manner which might instigate future change by institutions. Participants also described how Research afforded safety to participants in expressing their stories, especially for those seeking to debrief anonymously.

### 1a) Research provides an opportunity to voice, broadcast and enact change

“So, if your project…or thesis comes out and makes changes in even one person’s life, it will be better”[Interviewee #34; PMQ: India].

Several interviewees reported participating in the research study to effect change in institutional policies, identifying that publication of research would be a powerful tool to do so. One IMG explained: “A lot of countries are pretty unstable, and Australia remains a country that is highly desirable to practice in, so yeah, I think it’s so worth shining a light on this issue” [Interviewee #36; PMQ: South Africa].

Many interviewees mentioned the importance of Research as a medium to “convey a message” [Interviewee #09; PMQ: India], particularly from marginalised groups, or those who felt underrepresented. Publication was depicted as a means to project voices of those who otherwise felt powerless to speak, and broadcast issues to wider circles. A few interviewees also mentioned that sharing the study findings would be a way of supporting other IMGs facing adversity, encouraging them that they are not alone. One IMG explained: “we want our voice to be heard. We have been suffering a lot and its unfair… and no one knows” [Interviewee #19; PMQ: Italy].

### 1b) Safe and anonymous sharing of personal stories and experiences is therapeutic

“…thank you for giving me a chance…for telling you bad stories” [Interviewee #20; PMQ: Egypt].

Several interviewees reported the process of storytelling as important for expressing themselves, validating their feelings and as a therapeutic outlet. Interviewees spoke about the ‘therapy’ being connected to the anticipated hope for change. Several IMGs were audibly distressed during the interview, telling their story with a crying or angry voice. One IMG described “goosebumps” when recounting their story of bullying and other stressful life events. Interviewees described a sense of freedom in sharing personal stories within the safety of Research confidentiality, without fear of judgement or repercussion. Several interviewees reported not having the opportunity to share their full experiences or express their distress or concerns safely, until these interviews. One IMG disclosed: “I express myself a lot to you…rather than to my friend!” [Interviewee #26; PMQ: Bangladesh].

Interviewees explained that storytelling provided some meaning and validation of their experience. One interviewee explained that the interview process was necessary to explain details of their experience, which could not otherwise be captured: “because I didn’t have anyone to tell this to…. So, I wanted a space. I wanted to participate in something that my struggles and my experiences would have been heard…. And this can’t be written down. It has to be spoken, verbally…” [Interviewee #35; PMQ: Ireland].

## Theme 2: personal relevance and interest motivates participation

Interviewees reported that their interest in either the subject matter or the subject population triggered their desire to participate in the research.

### 2a) Passion for the research topic drives participation

“I’m really keen to see the results of this study. I just think…it’s such a worthy project… and a space that needs to be addressed.” [Interviewee #12; PMQ: UK].

Personal interest in the research topic was expressed by many interviewees. Some described their strong passion: “Because I think it’s really, really important that we do whatever we have to do, to make IMGs succeed.” [Interviewee #21; PMQ: UK]. Others explained how their personal experiences or observations sparked interest in the topic being studied, particularly those who had had traumatic or negative experiences. One interviewee explained that their anger with current bureaucratic dysfunction fuelled their desire to participate and tell their story by interview.

### 2b) Comradery and empathy for peers convicts participants to advocate for others through engagement

“I sort of felt like I needed to stand up for my colleagues…I see that my colleagues are being treated [poorly]… And I think that that is unfair.” [Interviewee #12; UK].

Many participants reported that their experiences or observations of challenge convicted them to participate in the study to help other/future IMGs. One interviewee simply responded: “…because I don’t want other IMGs [to] experience what I experienced. ” [Interviewee #25; PMQ: China].

Several interviewees spoke of their comradery for fellow IMGs and bonding over shared collective experiences. One IMG said: “I guess it’s a sense of comradery, in terms of the IMG community. We know we’ve gone through a lot and we’re still a long way away from getting what we want and where we want to be” [Interviewee #06; PMQ: Philippines]. Interviewees reported finding satisfaction in helping others. One IMG said: “ I really want to be helpful. Because people will be going through the same, similar journey….” [Interviewee #10; PMQ: India].

Interviewees expressed a sense of responsibility for IMGs in less fortunate situations. Several interviewees described a desire to raise their voice on behalf of colleagues who had experienced discrimination or oppression. In this way, the interviewees considered their participation as an opportunity to show solidarity for their peers. One IMG explained: “I was so inspired by the opportunity to encourage IMGs who were really, really suffering in the system.” [Interviewee #31; PMQ: UK]. Furthermore, at the conclusion of interviews, several interviewees openly offered to generously share their time and experience as a mentor or contact person for any new IMGs seeking advice.

## Theme 3: Respect for Research drives engagement

“I guess it wasn’t specific to being an IMG, I just like to help out with medical research.” [Interviewee #24; UK].

A few interviewees reported participating simply for the importance of supporting Research as an entity, and contributing to the scholarly realm. One interviewee participated to provide some variety to the study results, whilst others participated in empathy for the researcher. One IMG said: “…I know how difficult it is to interview people…. So, I have to encourage that person who is doing the study.” [Participant #18; PMQ: India].

## Discussion

Our findings indicated a variety of reasons why IMGs chose to participate in research. We found that the doctors in our study valued research as a trustworthy and powerful means of disseminating rigorous findings and enacting meaningful change for the future. Other reasons for participating included having an avid interest and empathy for the study topic or study population and a respect for Research and the furthering of science. Participant descriptions of a perceived lack of IMG advocacy, discrimination and the hope of change for the future were prominent within the data, findings which are explored more fully in our related published and unpublished works [13, 18]. 

We found that research in itself was viewed by IMGs in our study as a safe and acceptable method for sharing sensitive stories and viewpoints. Expressing the voice of minority groups [24, 25] and furthering of scientific knowledge have been identified as important factors for participant engagement in other studies [26, 27]. Indeed, lack of representation in studies may compromise generalizability and cultural validity of results [24, 28]. As researchers strive for equity and diversity in research, it is imperative that researchers are attentive to methodology which is acceptable to target populations [28, 29]. Wide engagement is needed to hear a range of stakeholder voices and perspectives.

The governances around research confidentiality was particularly reassuring for our participants who had otherwise not had the confidence nor opportunity to disclose and voice concerns elsewhere, without fear of repercussion or retribution. Worryingly, our findings highlight the degree of isolation felt by some IMGs and the absence of a safe outlet to disclose their private distress. This may reflect the low rates of established mentoring and peer support which have been observed by the authors in unpublished work. Mentorship and other peer support services are useful for providing a space for debriefing and accessing emotional support [30]. Other contributions to feelings of isolation may indicate a lack of informal social support networks or a lack of understanding of work rights in Australia. IMGs should be made aware of confidential services available in their host country, to support them with complaints processes and work-related challenges. For example, in Australia, services include the National Health Practitioner Ombudsman, medical indemnity services, the Doctors Health Services and employee assistance programs [31–33]. 

Indeed, the interest demonstrated by IMGs in participating in this study emphasizes the importance of this research topic for IMGs at both an individual and community level. Studies show that people are motivated to act as research participants where they have personal, therapeutic and material interests in the research study [34, 35]. 

The offer of financial incentives to study participants is controversial, with ethical concerns around undue coercion and recruiting bias [36]. Although financial incentive has been identified as a key motivator for healthy people choosing to participate in research [26]we chose against this in our study. Our participants received no financial reward, thereby adding to our view of the sample’s generosity in their voluntary contribution to this study. Altruism, informing change, and the therapeutic effects of interviewing were observed in our study, and have been supported by others conducting qualitative research [35, 37]. 

Lastly, our participants demonstrated empathy, moral duty and curiosity- elements distinctly reminiscent of our Hippocratic Oath. The show of comradery for others in the IMG community was remarkable. Indeed, the power of collectiveness and importance of social capital is evidenced by our participants across Australia who have shared their stories for this study.

### Strengths and limitations

Our study is valuable as it provides new insights into the motivations of doctors as research participants. Such insights may assist prospective researchers looking to explore health system, training and career issues from an IMG viewpoint. Furthermore, important IMG needs (e.g., the desire to enact change and the requirement for safe debriefing space) are identified and highlighted in this paper. However, there are limitations to our study, such as the propensity to sampling bias and the inability to generalize due to limited similar studies in the area. Nonetheless, the individual opinions expressed by the participants demonstrate the importance of listening to the IMG voice in future studies involving their training and career in Australia.

## Conclusion

Including IMGs in research is an important step to addressing issues in healthcare settings, as IMGs may provide valuable insights for innovative improvements to health system challenges. IMGs demonstrate a variety of personal motives for participating in research. Researchers may use this understanding to better engage IMGs as participants in future research endeavours.

## Electronic supplementary material

Below is the link to the electronic supplementary material.

Supplementary Material 1

## Data Availability

Data is provided within the manuscript or supplementary information files.

## References

[1] Lyng HB, Macrae C, Guise V, Haraldseid-Driftland C, Fagerdal B, Schibevaag L, Alsvik JG, Wiig S. Balancing adaptation and innovation for resilience in healthcare–a metasynthesis of narratives. BMC Health Serv Res. 2021;21:1–3. 34332581 10.1186/s12913-021-06592-0 PMC8325788

[2] Agency for Clinical Innovation [Internet]. St Leonards: NSW Government [cited 2024 Sep 13]. Available from: https://aci.health.nsw.gov.au/about/about-us

[3] Markwell S, Beynon C. Health service development and planning [Internet]. London: Faculty of Public Health; 2009. Chapter 5d, Understanding the Theory and Process of Strategy Development. [cited 2024 Sep 13]. Available from: https://www.healthknowledge.org.uk/public-health-textbook/organisation-management/5d-theory-process-strategy-development/health-service-development-planning

[4] Malterud K, Fredriksen L, Gjerde MH. When Doctors experience their vulnerability as beneficial for the patients: a focus-group study from general practice. Scand J Prim Health Care. 2009;27(2):85–90. 19137476 10.1080/02813430802661811 PMC3410467

[5] Kay M, Mitchell G, Clavarino A, Doust J. Doctors as patients: a systematic review of doctors’ health access and the barriers they experience. Br J Gen Pract. 2008;58(552):501–8. 18611318 10.3399/bjgp08X319486 PMC2441513

[6] Morishita M, Iida J, Nishigori H. Doctors’ experience of becoming patients and its influence on their medical practice: A literature review. Explore. 2020;16(3):145–51. 31843394 10.1016/j.explore.2019.10.007

[7] Horowitz CR, Suchman AL, Branch WT, et al. What do doctors find meaningful about their work?? Ann Intern Med. 2003;138:772–5. 12729445 10.7326/0003-4819-138-9-200305060-00028 PMC4303370

[8] Dopelt K, Bachner YG, Urkin J, Yahav Z, Davidovitch N, Barach P. Perceptions of practicing physicians and members of the public on the attributes of a good doctor. InHealthcare 2021 Dec 31 (Vol. 10, No. 1, p. 73). MDPI. 10.3390/healthcare10010073 PMC8775310 35052237

[9] Morrow G, Burford B, Carter M, Illing J. Have restricted working hours reduced junior doctors’ experience of fatigue? A focus group and telephone interview study. BMJ Open. 2014;4(3):e004222. 10.1136/bmjopen-2013-004222 PMC3948452 24604482

[10] Gregory S, Demartini C. Satisfaction of Doctors with their training: evidence from UK. BMC Health Serv Res. 2017;17:1–8. 29284467 10.1186/s12913-017-2792-0 PMC5747190

[11] Cuesta-Briand B, Coleman M, Ledingham R, Moore S, Wright H, Oldham D, Playford D. Understanding the factors influencing junior doctors’ career decision-making to address rural workforce issues: testing a conceptual framework. Int J Environ Res Public Health. 2020;17(2):537. 31952128 10.3390/ijerph17020537 PMC7013936

[12] Healey SJ, Fakes K, Nair BR. Inequitable treatment as perceived by international medical graduates (IMGs): a scoping review. BMJ Open. 2023;13(7):e071992. 10.1136/bmjopen-2023-071992 PMC10347491 37438072

[13] Healey SJR, Fakes K, Malau-Aduli B, Leigh L, Nair BR. Exploring experiences of work-related inequitable treatment among international medical graduates (IMGs): a sequential explanatory mixed methods study. PLoS ONE. 2025;20(2):e0319230. 39982957 10.1371/journal.pone.0319230 PMC11845036

[14] International Medical graduates (IMGs) [Internet], Medical Board AHPRA. 2024 [cited 2024 July 31]; Available from: Medical Board of Australia - International medical graduates (IMGs).

[15] Tran M, Lee JT, Cormack M et al. Health Workforce Shortage in Australia Post COVID-19- International Perspectives. Australian National University. National Centre for Health Workforce Studies: Health Workforce Data Insights- Mar 2024. Accessed 10 July 2024. NCHWS Data Insight Serries - Mar2024.pdf (anu.edu.au).

[16] Foreign-Trained Doctors are Critical to Serving Many U.S. Communities. Special Report Jan 2018. American Immigration Council. Accessed 14 July 2024. foreign-trained_doctors_are_critical_to_serving_many_us_communities.pdf (americanimmigrationcouncil.org).

[17] The state of medical education and practice in the UK: Workforce Report. 2023. General Medical Council. London (UK). Accessed 14 July 2024. *UK Workforce-report-2023-full-report_pdf-103569478.pdf.

[18] Healey SJ, Fakes K, Malau-Aduli BS, Nair BR. A Psychosocial Exploration of International Medical Graduate Journeys, Perceptions, Challenges and Resulting Impacts: A Sequential Explanatory Mixed Methods Study. Advances in Medical Education and Practice. 2025 May 31;16:965-79 https://www.dovepress.com/articles.php?article_id=103479 10.2147/AMEP.S521037 PMC12135948 40470264

[19] Australian Parliament House of Representatives Standing Committee of Health and Ageing. 2012. Lost in the labyrinth: report on the inquiry into registration processes and support for overseas trained doctors Available at: https://www.aph.gov.au/parliamentary_business/committees/house_of_representatives_committees?url=haa/overseasdoctors/report.htm

[20] Lloyd T, Phillips BR, Aber RC. Factors that influence doctors’ participation in clinical research. Med Educ. 2004;38(8):848–51. 15271045 10.1111/j.1365-2929.2004.01895.x

[21] Braun V, Clarke V. Using thematic analysis in psychology. Qualitative Res Psychol. 2006;3(2):77–101.

[22] NVivo 14- Leading Qualitative Data Analysis Software with AI Solution. Lumivero. 2024. Accessed 10 July 2024. NVivo - Lumivero.

[23] Mann K, MacLeod A. Constructivism: learning theories and approaches to research. Researching medical education. Chichester, UK: John Wiley & Sons, Ltd; 2015. pp. 49–66.

[24] Redwood S, Gill PS. Under-representation of minority ethnic groups in research—call for action. Br J Gen Pract. 2013;63(612):342–3. 23834862 10.3399/bjgp13X668456 PMC3693777

[25] Healey SJ, Ghafournia N. Lessons from pandemic research with refugee communities. Public Health Res Pract. 2022;32(4). 10.17061/phrp3242236 36509685

[26] Stunkel L, Grady C. More than the money: a review of the literature examining healthy volunteer motivations. Contemp Clin Trials. 2011;32(3):342–52. 21146635 10.1016/j.cct.2010.12.003 PMC4943215

[27] Alexander SJ. As long as it helps somebody’: why vulnerable people participate in research. Int J Palliat Nurs. 2010;16(4):173–8. 20559179

[28] Leong FT, Kalibatseva Z, Scott NA. Dismantling racism in research methods and practices. Rev Gen Psychol. 2024;28(4):301–14.

[29] Roberts SO, Bareket-Shavit C, Dollins FA, Goldie PD, Mortenson E. Racial inequality in psychological research: trends of the past and recommendations for the future. Perspectives on psychological science. 2020 Nov;15(6):1295-309. 10.1177/1745691620927709 32578504

[30] Henry-Noel N, Bishop M, Gwede CK, Petkova E, Szumacher E. Mentorship in medicine and other health professions. J Cancer Educ. 2019;34:629–37. 29691796 10.1007/s13187-018-1360-6

[31] Anonymous and confidential complaints [Internet]. Melbourne: The National Health Practitioner Ombudsman; 2024 [cited 2024 Sep 13]. Accessible from: https://www.nhpo.gov.au/anonymous-and-confidential-complaints

[32] Getting help [Internet]. Brisbane: Doctors Health Services; 2024 [cited 2024 Sep 14]. Accessible from: https://www.drs4drs.com.au/getting-help

[33] Employee Assistance Program [Internet]. EAPassist. 2008 [cited 2024 Sep 9]. Accessible from: https://eapassist.com.au/?gclid=EAIaIQobChMI8rnpyIi1iAMVX1kPAh1ggBmjEAAYAiAAEgKKvPD_BwE

[34] Keusch F. Why do people participate in web surveys? Applying survey participation theory to internet survey data collection. Manage Rev Q. 2015;65(3):183–216.

[35] Clark T. On ‘being researched’: why do people engage with qualitative research? Qualitative Res. 2010;10(4):399–419.

[36] Resnik DB. Bioethical issues in providing financial incentives to research participants. Medicolegal and bioethics. Jun. 2015;24:35–41. 10.2147/MB.S70416 PMC4719771 26807399

[37] Peel E, Parry O, Douglas M, Lawton J. It’s no skin off my nose: why people take part in qualitative research. Qual Health Res. 2006;16(10):1335–49. 17079797 10.1177/1049732306294511

