# Supplementary material for: Doctors as research participants - what is in it for them? A qualitative study of international medical graduates
Source: BMC Med Educ. 2025 Jul 1;25:878. doi: 10.1186/s12909-025-07468-1 (PMC12210775; doi:10.1186/s12909-025-07468-1)
Supplement: Supplementary file 1 — Supplementary Material 1 [file 12909_2025_7468_MOESM1_ESM.pdf]

Semi-structured interview question for qualitative study

From our survey, we found the majority of participants wanted to be interviewed, which we felt is a finding worth investigating in itself. Why did you choose to participate in this research?<br>(Probe: motivations, stimulus, How do you think this process of research has been beneficial to you?)
